# Isolated diastolic potentials as predictors of success in ablation of right ventricular outflow tract idiopathic premature ventricular contractions

**DOI:** 10.1371/journal.pone.0211232

**Published:** 2019-02-06

**Authors:** Leonor Parreira, Rita Marinheiro, Pedro Carmo, Pedro Amador, Dinis Mesquita, José Farinha, Diogo Cavaco, Rafael Jeronimo, Francisco Costa, Pedro Adragão

**Affiliations:** 1 Arrhythmology Department, Hospital da Luz, Lisboa, Portugal; 2 Cardiology Department, Centro Hospitalar de Setúbal, Setúbal, Portugal; University of Minnesota, UNITED STATES

## Abstract

**Background and aims:**

Discrete potentials, low voltage and fragmented electrograms, have been previously reported at ablation site, in patients with premature ventricular contractions (PVCs) originating in the right ventricular outflow tract (RVOT). The aim of this study was to review the electrograms at ablation site and assess the presence of diastolic potentials and their association with success.

**Methods:**

We retrospectively reviewed the electrograms obtained at the radiofrequency (RF) delivery sites of 48 patients subjected to ablation of RVOT frequent PVCs. We assessed the duration and amplitude of local electrogram, local activation time, and presence of diastolic potentials and fragmented electrograms.

**Results:**

We reviewed 134 electrograms, median 2 (1–4) per patient. Success was achieved in 40 patients (83%). At successful sites the local activation time was earlier– 54 (-35 to -77) ms vs -26 (-12 to -35) ms, p<0.0001; the local electrogram had lower amplitude 1 (0.45–1.15) vs 1.5 (0.5–2.1) mV, p = 0.006, and longer duration 106 (80–154) vs 74 (60–90) ms, p<0.0001. Diastolic potentials and fragmented electrograms were more frequently present, respectively 76% vs 9%, p <0.0001 and 54% vs 11%, p<0.0001. In univariable analysis these variables were all associated with success. In multivariable analysis only the presence of diastolic potentials [OR 15.5 (95% CI: 3.92–61.2; p<0.0001)], and the value of local activation time [OR 1.11 (95% CI: 1.049–1.172 p<0.0001)], were significantly associated with success.

**Conclusion:**

In this group of patients the presence of diastolic potentials at the ablation site was associated with success.

## 1. Introduction

The most common site of premature ventricular contractions (PVCs), in patients without structural heart disease, is the right ventricular outflow tract (RVOT) [1]. The intracardiac bipolar ventricular electrograms in the absence of structural heart disease, typically show a sharp deflection with normal amplitude and duration. Ablation based on activation mapping and/or pace-mapping is considered the standard technique for eliminating idiopathic PVCs arising from the RVOT [2]. Radiofrequency delivery should be performed at the site of the earliest ventricular activation. The ideal pace-map at ablation site is an identical QRS pattern in all 12 surface ECG leads (12/12 match).

Despite the high success rate [3] there are cases when complete eradication of the PVCs cannot be obtained. Many authors have looked for potential predictors of success, clinical and electrocardiographic or based in electrophysiological findings [4–6].

In our paper, we describe the presence of discrete isolated diastolic potentials, on the bipolar intracardiac electrograms at ablation site. These are low amplitude potentials, occurring after the T wave of the ECG in sinus rhythm that become pre-systolic, preceding the local bipolar ventricular electrogram during the PVCs. The meaning of these isolated diastolic potentials is unknown, and the aim of the present study was to evaluate their prevalence during RVOT PVCs ablation and their impact on the success of the procedure.

## 2. Methods

### 2.1. Patient selection

We studied 48 patients subjected to catheter ablation of idiopathic RVOT PVCs, between January 2010 and January 2018 in two Hospitals.

All patients underwent transthoracic echocardiography, including 2-dimensional, M-mode, and Doppler echocardiography as well as 12-lead electrocardiograms (ECG). Whenever a structural heart disease was suspected, due to the presence of electrocardiographic or echocardiographic anomalies, the presence of syncope or a family history of sudden death, a cardiac magnetic resonance imaging was performed. When symptoms were triggered or aggravated by exercise, a treadmill stress test was performed to rule out ischemia and, in case of doubt, a computed tomography angiography was done to exclude coronary artery disease. Cardiac magnetic resonance imaging was also performed at the discretion of the attending physician.

Patients were excluded if a structural heart disease was present, if the patient had been subjected to previous ablation or if the PVCs focus was outside the RVOT.

### 2.2. Study design

We retrospectively analyzed all intracardiac electrograms at the site of RF delivery, to assess the presence of diastolic potentials. Other characteristics of the local bipolar electrogram were evaluated, including the local activation time in relation to the beginning of the QRS of the PVCs on the surface ECG, their amplitude, duration and the presence of fragmented electrograms. The electrograms were evaluated by two senior electrophysiologists. For the purpose of this study we only considered RF applications that lasted at least 60 seconds.

We evaluated the association between the presence of diastolic potentials and procedure acute success rate, adjusted to the other variables.

### 2.3. Mapping and ablation

#### 2.3.1. Mapping technique and measurements

Patients were studied in a fasting non-sedate state. All beta-blockers and antiarrhythmic drugs were discontinued at least five half-lives before the electrophysiological study.

Diagnostic catheters were positioned via the femoral vein with fluoroscopic guidance in the His position and in the great cardiac vein via the coronary sinus.

Isoprenaline was administered intravenously, as needed, and titrated to a dose capable of inducing PVCs.

All patients underwent electroanatomical mapping by the CARTO 3 system (Biosense-Webster) or EnSite Velocity system (Abbott).

With the CARTO 3 system all procedures were performed using the Niobe magnetic navigation system (Stereotaxis) working with the monoplane fluoroscopy system AXIOM Artis (Siemens) as previously described by Parreira et al [7]. An irrigated tip Navistar RMT Thermocool catheter (Biosense-Webster) was used with a 3.5-mm distal tip electrode and a 2-5-2 interelectrode distance.

With the EnSite Velocity system all procedures were done manually with the monoplane fluoroscopy system BV Pulsera (Philips) and using an irrigated tip Therapy Cool Path or Flexability catheter (Abbott) with a 4-mm distal tip electrode and a 1-4-1 interelectrode distance.

The ablation catheter was introduced via the femoral vein, manually advanced to the right atrium and then automatically advanced to the His bundle and RVOT in the magnetic navigation system patients or manually in the EnSite patients, under fluoroscopic guidance. The ablation catheter was then placed at multiple sites on the endocardial surface of the RVOT. The 12-lead surface ECGs and intracardiac electrograms were recorded simultaneously by a digital multichannel system, filtered at 30–300 Hz for bipolar electrograms and at 0.05–525 Hz for unipolar electrograms, displayed at 100 mm/s speed. The bipolar electrograms were analyzed in regard of their timing in relation to the onset of the QRS on the surface ECG, their local amplitude, duration and presence of multiple components. The information was used to generate 3-dimensional electroanatomical activation and voltage maps of the RVOT, with the electrophysiologic information, color coded and superimposed on the geometry. The color display for voltage mapping ranged from purple, representing electroanatomical normal tissue (amplitude > 1.5 mV), to red, representing electroanatomical scar tissue (amplitude <0.5 mV). Intermediate colors represented regions with electroanatomical low voltage.

#### 2.3.2. Diastolic potentials

**Fig 1:**
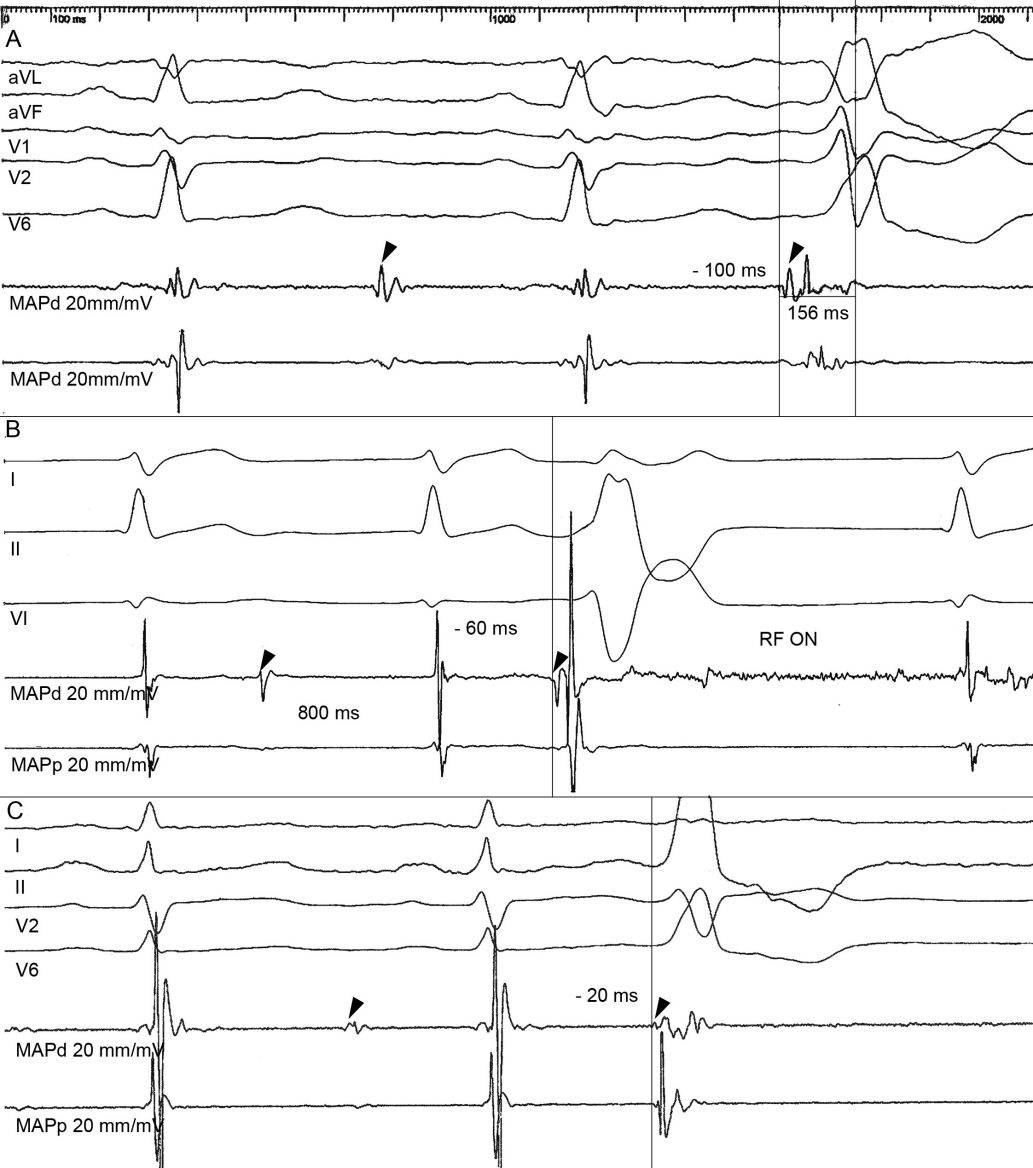
Diastolic potentials. Representative intracardiac electrograms at a successful ablation sites of 3 different patients. The MAPd exhibits the diastolic potentials (arrow head), occurring after the T wave of the surface ECG in sinus rhythm, becoming pre-QRS during the PVCs. The gain in the ablation catheter is 20 mm/1mV and sweep speed is 100 mm/sec. (A), fragmented diastolic potential preceding the QRS by -100 ms; (B) sharp diastolic potential preceding the QRS by—60 ms. (C) dull diastolic potential preceding the QRS by -20 ms. In panel B and C the local electrogram in sinus rhythm displays normal amplitude and duration. In Panel A the local electrogram shows low voltage and prolonged duration.

Diastolic potentials were defined as persistent low amplitude discrete potentials occurring at late diastole, after the end of the T wave of the surface ECG in sinus rhythm. These diastolic potentials became presystolic during the PVCs and were only recorded close to the ablation site (Fig 1).

#### 2.3.3. Fragmented electrograms

**Fig 2:**
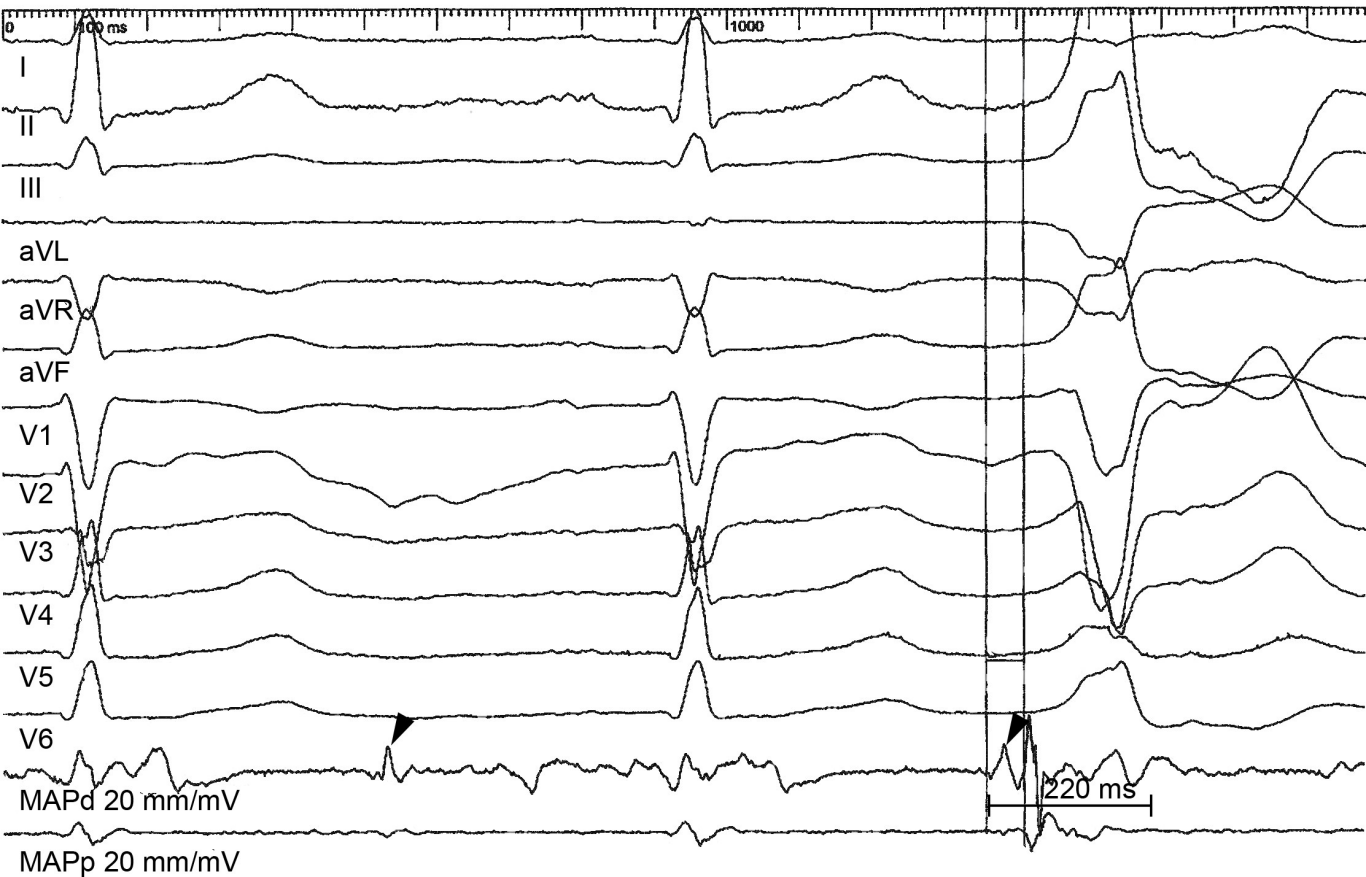
Fragmented electrograms. Intracardiac electrograms at successful ablation site. The bipolar electrogram on the ablation catheter (MAPd) exhibits a typical fragmented electrogram, diastolic potentials are also present (arrow heads). The fragmented electrogram shows low voltage and prolonged duration, 220 ms, but ending before the end of the QRS.

Defined as bipolar electrograms at ablation site, with low amplitude, long duration and multiple peaks (Fig 2).

#### 2.3.4. Activation mapping and ablation

The activation map was created by mapping several points within the RVOT during each PVCs while using a surface ECG lead as reference. Activation times were assigned based on the onset of bipolar electrograms. After isochronal reconstructions of the RVOT were generated, bipolar pace mapping was performed at multiple endocardial sites near the earliest activation site. Pacing was performed at cycle lengths as close as possible to that of the coupling interval of the PVCs.

The ablation site was selected based on the earliest endocardial activation time with a QS pattern at the unipolar electrogram and confirmed by the pace mapping that provided at least a 11 out of 12 match between paced and spontaneous PVCs.

Energy was delivered from an EP Shuttle RF generator (Stockert) between the distal electrode of the ablation catheter and a cutaneous patch, for up to 120 seconds, to a maximum temperature of 43°C and a power output limit of 40 W. When the application was ineffective, additional applications were delivered to sites adjacent to the earliest activation site displaying a good pace-map matching. During ablation, light sedation with midazolam (bolus) or remifentanil (continuous perfusion) was administered when needed.

Success was defined as abolition of PVCs under isoprenaline infusion until thirty minutes after ablation.

All patients were monitored in hospital for 24 hours after the procedure.

### 2.4. Follow-up

Follow-up was performed on outpatient clinical visits. Clinical assessment was performed 1 to 3 months after ablation and regularly every 6 months thereafter. In patients that were followed by another physician the contact was performed by phone. All patients had a 24-hour Holter recording done after the procedure.

### 2.5. Statistical analysis

SPSS version 23 software (SPSS Inc., Chicago, Illinois) was used for statistical analysis. Data is expressed as median and interquartile range for continuous variables and as frequencies and percentages for categorical variables. Baseline characteristics were compared using the chi-square test for categorical variables and the Mann-Whitney-U test for continuous variables. Univariable and multivariable logistic regression analysis was used to calculate the odds ratios (OR) and 95% confidence intervals (CI). A value of p < 0.05 was considered statistically significant.

### 2.6. Ethics

All patients signed the informed consent form and the study was approved by the Ethics Committee for Health of Hospital da Luz and Ethics Committee for Health of Centro Hospitalar de Setúbal. The study is in compliance with the Helsinki Declaration.

## 3. Results

### 3.1. Study population

We reviewed 134 electrograms from forty-eight patients that entered the study, median age was 40 (31–56) years, eighteen males. Patient characteristics are displayed on Table 1.

**Table 1:** Baseline characteristics of the studied patients.

	Overall sample (n = 48)	Successful procedure (n = 40)	Unsuccessful procedure (n = 8)	P value^a^
**Demographic data**				
Age (years)	40 (31–56)	40 (33–50)	53(19–61)	0.609
Male gender, n (%)	18 (38)	13 (33)	5 (62)	0.132
**Risk factors and history**				
Family history of sudden death, n (%)	2 (4)	2 (5)	0	0.9999
Absence of risk factors, n (%)	39 (81)	34 (85)	5 (63)	0.330
Strenuous exercise, n (%)	5 (10)	3 (8)	2 (25)	0.189
**Symptoms**				
Asymptomatic, n (%)	4 (8)	2 (5)	2 (25)	0.124
Syncope/near syncope n (%)	7 (14)	5 (13)	2 (25)	0.330
Palpitations, n (%)	43 (89)	37 (93)	6 (75)	0.189
**Medications**				
Betablockers, n (%)	24 (50)	21 (53)	3 (38)	0.701
Antiarrhythmics, n (%)	11 (23)	8 (20)	3 (38)	0.361
**12 Lead ECG**				
T wave inversion after V1, n (%)	8 (17)	6 (15)	2 (25)	0.605
**Treadmill stress test (n = 28)**				
Exercise induced increase in PVCs frequency, n (%)	6 (21)	6 (15)	0	0.542
**24-Hour Holter recording**				
N° of PVCs/24 hours	18250 (15000–24000)	18000 (15000–24000)	19250 (15750–23000)	0.857
NSVT n (%)	6 (12)	6 (15)	0	0.571
**Mapping data**				
Mapping system (CARTO/EnSite), n	35/13	29/11	6/2	0.9999
EAS RVOT free wall, n (%)	15 (31)	11 (27)	4 (50)	0.236
EAS RVOT septum, n (%)	33 (69)	29 (73)	4 (50)	0.236
Number of points in the map	67 (50–94)	64 (50–94	70 (52–95)	0.782
Number of RF pulses	2 (1–4)	2 (1–3.75)	4.5 (4–6)	0.0001

Values are presented as median (interquartile range) and number (%). EAS, earliest activation site; NSVT, non-sustained ventricular tachycardia; PVCs, premature ventricular contraction; RF, radiofrequency; RVOT, right ventricular outflow tract.

^a^ p values were calculated using Mann-Whitney-U test for continuous variables and the chi-square test for categorical variables.

Only four patients were asymptomatic while almost 90% of patients complained of palpitations. Seven patients had a history of syncope or near syncope, although none had documented episodes of sustained ventricular arrhythmias. Two patients had family history of sudden death, but none had family history of inherited arrhythmic disorders. Twenty-four patients were on beta-blockers and eleven were on antiarrhythmics other than amiodarone. Physical examination and transthoracic echocardiography, including 2-dimensional, M-mode, and Doppler echocardiography were normal and demonstrated normal right ventricle size and function in all patients. The ECG displayed T wave inversion beyond V1 in 8 patients but arrhythmogenic right ventricular cardiomyopathy was ruled out according to the 2010 Task Force Criteria [8]. Twenty-eight patients underwent treadmill stress test that showed a reduction of the PVCs frequency in twenty-two (79%) patients and an increase in six (21%), without evidence of ischemia. Three patients underwent computed tomography angiography, with normal results. Fifteen patients underwent cardiac magnetic resonance imaging, without any relevant findings. A flecainide test was performed in one patient with ST elevation and syncope to rule out Brugada Syndrome. The 24-hour Holter recording showed a high PVCs burden with a median 18250 (15000–24000) PVCs/24 hours and the occurrence of runs of non-sustained ventricular tachycardia in six patients. The demographic and clinical characteristics did not differ whether the procedure was successful or unsuccessful.

### 3.2. Mapping and ablation

#### 3.2.1. Activation mapping and ablation

The median number of acquired points in the RVOT were 67 (50–94). The activation map identified the earliest activation site in the RVOT free wall in fifteen patients and in the RVOT septum in thirty-three. The median number of RF pulses was 2 (1–4). The acute success rate was 83% and there were no complications (Table 1). In the forty patients with a successful procedure, thirty (75%) had diastolic potentials at ablation site and ten (25%) did not. Success was achieved in all thirty patients with diastolic potentials and only in 56% of patients without. Forty-one RF pulses, out of the 134 analysed, were considered successful because they led to elimination of the PVCs. On one application the PVCs recurred after 30 minutes and a new RF pulse was applied at the same site with success (Table 2).

**Table 2:** Mapping and ablation data.

	**Overall sample (n = 134)**	**Successful** **(n = 41)**	**Unsuccessful** **(n = 93)**	**P value**^a^
**Ablation data**				
LAT (ms)	-30 (-20 to -44)	- 54 (-35 to -77)	- 27 (-16 to-38)	<0.0001
Amplitude of local electrogram (mV)	1 (0.5–2)	1 (0.45–1.15)	1.5 (0.5–2.1)	0.006
Duration of local electrogram (ms)	80 (64–100)	106 (80–154)	74 (60–90)	<0.0001
Presence of diastolic potentials, n (%)	39 (29)	31 (76)	8 (9)	<0.0001
Presence of fragmented electrograms, n (%)	32 (24)	22 (54)	10 (11)	<0.0001
	**Overall sample** **(n = 134)**	**With DP** **(n = 39)**	**Without DP** **(n = 95)**	**P value**^**a**^
**Ablation data**				
LAT (ms)	-30 (-20 to -44)	- 54 (-34 to -74)	- 27 (-16 to -38)	<0.0001
Amplitude of local electrogram (mV)	1 (0.5–2)	1 (0.5–1.5)	1.1 (0.5–2)	0.037
Duration of local electrogram (ms)	80 (64–100)	120 (80–160)	74 (60–90)	<0.0001
Presence of fragmented electrograms, n (%)	32 (24)	21 (54)	11(12)	<0.0001
Success, n (%)	40 (30)	31 (80)	10 (11)	<0.0001

Values are presented as median (interquartile range) and number (%). DP, diastolic potentials; LAT: local activation time.

^a^ p values were calculated using Mann-Whitney-U test for continuous variables and the chi-square test for categorical variables;

Successful RF applications had earlier local activation times—54 ms (-35 to -77) when compared to unsuccessful ones– 27 ms (-16 to-38). Diastolic potentials and fragmented electrograms were both more frequently present at successful sites, respectively 76% and 54% vs 9% and 11% at unsuccessful sites.

Comparing sites with diastolic potentials versus sites without diastolic potentials, the local activation time was earlier,– 54 (-34 to -74) ms vs -27 (-16 to -38) ms, p<0.0001, the median amplitude of the local electrogram was significantly lower, 1 (0.5–1.5) vs 1.1 (0.5–2) mV, p = 0.037 and the duration of the ventricular electrogram was longer, 120 (80–160) vs 74 (60–90) ms, p<0.0001. Fragmented electrograms were more frequently present at sites with diastolic potentials, (54% vs 12%; p<0.0001).

When analysing with univariable logistic regression the association between the analysed ablation parameters and the acute success, we found that all parameters were associated with success. In multivariable analysis we found that only the value of the local activation time and the presence of diastolic potentials were independently associated with success. The preliminary main effects analysis for both variables showed a 11% increase in the possibility of success for each ms of earliness of local activation, [OR 1.11 (95% CI: 1.049–1.172 p<0.0001)]. The presence of diastolic potentials was associated with a sixteen times higher possibility of success [OR 15.5 (95% CI: 3.92–61.2; p<0.0001)] (Table 3).

**Table 3:** Univariable and multivariable logistic regression analysis.

	Univariable analysis		Multivariable analysis	
	OR (95% CI)	P value^a^	OR (95% CI)	P value^a^
LAT (ms)	1.123 (1.075–1.173)	<0.0001	1.11 (1.049–1.172)	<0.0001
Amplitude of local electrogram (mVx10^-1^)	0.940 (0.899–0.982)	0.006	0.949 (0.899–1.028)	0.198
Duration of local electrogram (ms)	1.031 (1.017–1.044)	<0.0001	0.996 (0.974–1.017)	0.692
Presence of diastolic potentials	32.9 (11.9–91)	<0.0001	15.5 (3.92–61.2)	<0.0001
Presence of fragmented electrograms	9.6 (3.9–23.6)	<0.0001	1.707 (0.296–9.848)	0.550

CI: confidence interval; LAT: local activation time; OR: odds ratio

^a^ p values were calculated using univariable and multivariable logistic regression analysis

However, these main effects are qualified by an interaction between both variables. The presence of diastolic potentials reduces the OR for the local activation time from 1.11 to 1.061, meaning that in the presence of diastolic potentials the earliness of the local activation is less important to achieve success. The OR for the presence of diastolic potentials decreases with the increasing earliness of local activation and varies from OR 52.45 for local activation time of -30 ms, OR 12.8 for local activation time of -40 ms, OR 3.1 for local activation time– 50 ms, with no effect for local activation times earlier than this value.

#### 3.2.2. Diastolic potentials

The characteristics of the thirty-nine diastolic potentials present at RF application sites in thirty patients are presented in Table 4. In seven patients more than one RF application site displayed diastolic potentials.

**Table 4:** Characteristics of the diastolic potentials at RF delivery site.

Patient (RF	DP amplitude	QRS-DP (ms)	PVC	SR	Success	Morphology	Local electrogram
pulse)	(mV)		DP–QRS (ms)	DP-QRS (ms)	RF pulse		amplitude (mV)
1(1)	0.2	400	128	500	yes	Fragmented	1.3
2(1)	0.2	280	100	800	yes	Sharp	3.5
4(5)	0.1	380	10	300	yes	Dull	0.5
5(1)	0.1	360	94	430	yes	Dull	1
7(2)	0.1	400	74	450	yes	Dull	1
9(1)	0.1	400	72	400	yes	Fragmented	2
11(1)	0.1	400	60	500	yes	Fragmented	1.1
12(3)	0.1	360	10	330	no	Dull	1
12(4)	0.1	360	10	380	no	Dull	0.3
12(5)	0.1	360	52	300	yes	dull	0.3
14(1)	0.5	360	32	500	yes	Fragmented	3
17(1)	0.2	300	26	320	yes	Fragmented	0.6
21(2)	0.6	220	50	340	yes	Sharp	1.5
22(1)	0.3	340	64	380	yes	Fragmented	0,4
23(1)	0.2	400	40	400	no	Sharp	2.2
23(2)	0.3	400	30	500	no	Sharp	1.5
23(3)	0.1	400	96	450	yes	Sharp	0.2
24(2)	0.1	360	70	360	no	Dull	1.7
24(4)	0.1	360	80	400	yes	Dull	0.5
25(2)	0.2	320	80	350	yes	Fragmented	1
27(2)	0,1	400	30	420	yes	Fragmented	1
29(3)	0.6	400	42	320	no	Sharp	0,5
29(4)	0.5	400	52	420	yes	Sharp	0.4
30(2)	0.2	180	60	200	yes	Dull	1
32(1)	0.3	420	100	400	yes	Dull	1.5
33(1)	0.2	240	60	450	yes	Sharp	1.2
35(2)	0.6	400	60	360	yes	Sharp	0.5
35(3)	0.3	400	14	320	yes	Sharp	0.5
38(3)	0.1	380	60	300	yes	Dull	0.3
39(2)	0.1	300	55	260	yes	Dull	0,5
41(1)	0.1	300	100	200	yes	Dull	0.5
42(1)	0.1	360	25	400	no	Dull	1
42(5)	0.1	360	100	400	yes	Dull	0,2
43(1)	0.1	200	22	200	no	Dull	2
43(2)	0.1	200	30	200	yes	Dull	1
44(4)	0.1	350	100	650	yes	Fragmented	2
45(4)	0.5	400	64	320	yes	Dull	0,1
46(1)	0.2	300	20	350	yes	Fragmented	0,4
48(1)	0.1	350	20	400	yes	Dull	0.5

DP: diastolic potential; QRS-DP: interval between the end of the previous sinus QRS and the DP; PVC DP-QRS: interval between the DP and the QRS of the PVC; SR DP-QRS: interval between the DP and the QRS in sinus rhythm; PVC: premature ventricular contraction; RF: radiofrequency; SR: sinus rhythm.

**Fig 3:**
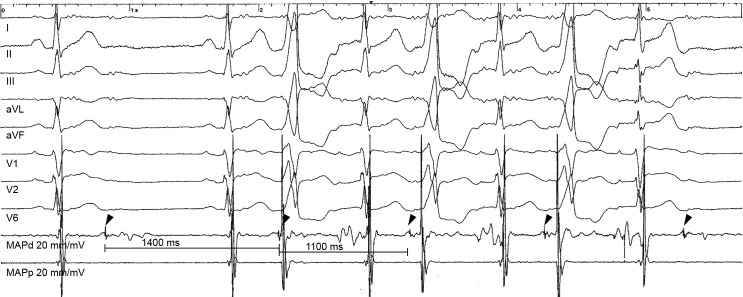
Variation in the inter diastolic potentials interval leading to a variation in the inter PVC interval. Intracardiac electrogram at successful ablation site. The bipolar electrogram on the ablation catheter (MAPd) exhibits sharp diastolic potentials (arrow head) after the T wave of the surface ECG in sinus rhythm, becoming pre-QRS during the PVCs. The variation of the interval between consecutive diastolic potentials is accompanied by a variation in the interval between the consecutive PVCs.

None of these sites were above the pulmonary valve. The diastolic potentials varied in morphology, either fragmented, sharp or dull potentials (Fig 1), but they all had very low voltage, median 0.1 mV (0.1–0.3). The coupling interval to the end of the previous QRS was variable, but always after the end of the T wave in the surface ECG, median 360 ms (300–400). There was some irregularity in the inter diastolic potential interval that caused a similar variation in the interval between consecutive PVCs (Fig 3).

The interval between the diastolic potentials and the beginning of the QRS during PVCs varied from patient to patient and, in the same patient, from site to site. The diastolic potentials at successful sites were significantly earlier -60 ms (-31-94) versus -30 ms (-16-51); p = 0.016 when compared to unsuccessful sites.

**Fig 4:**
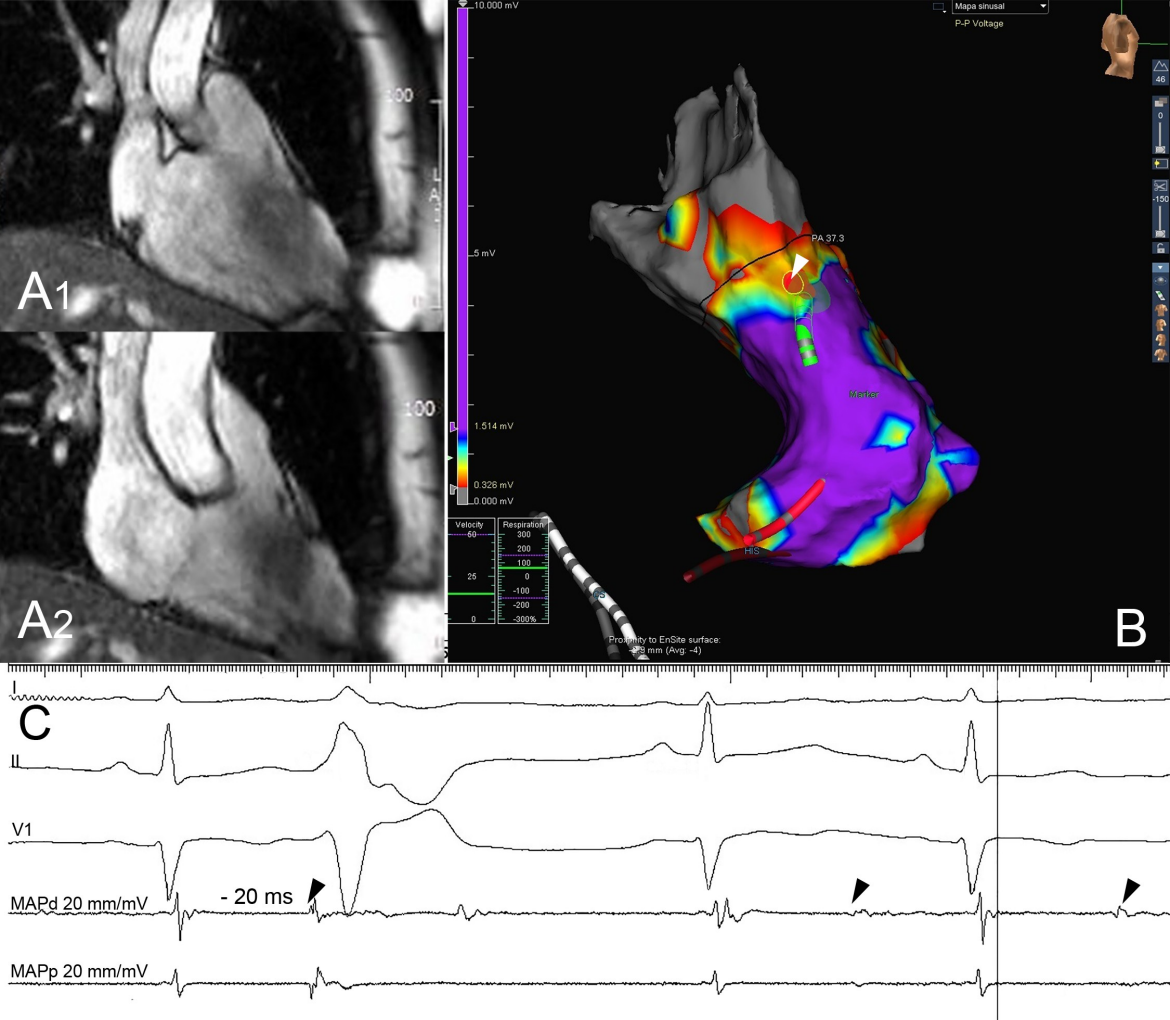
Low voltage areas. 35 years old female (patient 48) showing abnormal electrograms at ablation site despite apparent absence of structural heart disease. (A) Cardiac magnetic resonance steady-state free-procession (SSFP) cine imaging in the in-out view of the right ventricle in diastole (A1) and systole (A2) showing no abnormalities. (B) Electroanatomical voltage map in sinus rhythm. The black line indicates the pulmonary valve. The voltage map showing a wide area of low voltage below the pulmonary valve. The color map of voltage signals is explained in the methods. Purple indicates normal tissue while red indicates scar. Decapolar catheter in the coronary sinus and the His bundle catheter are displayed in white and red, respectively, at the bottom of the figure. The ablation catheter at the ablation site is indicated by the white arrow head. One RF ablation at this site caused the disappearance of the PVCs, indicated by the red dots. (C) The bipolar electrogram on the ablation catheter (MAPd) at successful ablation site exhibits very low voltage electrograms and dull diastolic potentials (black arrow heads) in sinus rhythm preceding the QRS by -20 ms during the PVC.

Diastolic potentials were mostly present in areas of low voltage with the median local electrogram voltage in sinus rhythm being 1 mV (0.5–1.5). Despite the presence of low voltage areas, patients had apparently normal hearts, with normal cardiac magnetic resonance imaging (Fig 4).

All patients with diastolic potentials underwent a successful procedure, although success was not obtained in the first site in seven patients. This was probably because at these unsuccessful sites the diastolic potential was not early enough in relation to the beginning of the QRS. Further mapping at this zone lead to the finding of an adjacent site with an earlier diastolic potential, where RF application was successful. During RF energy delivery we observed the disappearance of the diastolic potentials in a minority of cases (20%), preceded by a progressive reduction in the amplitude.

### 3.3. Follow-up

During a median follow-up of 48 months (32–80), there were 4 recurrences, all within the first year. Antiarrhythmic drugs were added to these patients, with symptomatic improvement. The other thirty-five patients remained asymptomatic with Holter recordings after the procedure showing a median of 5 PVCs/24 hours (1–30).

## 4. Discussion

The most important finding in this study was the recording of isolated diastolic potentials on the intracardiac bipolar electrogram, at successful ablation sites, during sinus rhythm. They were always found within the RVOT, below the pulmonary valve, and were only recorded in a small area around the successful ablation site. These diastolic potentials became pre-systolic during the PVCs.

Discrete isolated pre-systolic potentials have been previously described in PVCs originating above the pulmonary valve. Timmermans et al [9] described, for the first time, the occurrence of PVCs successfully ablated in the pulmonary artery. In five out of six patients they recorded a sharp local potential preceding the QRS of the PVCs, occurring late, after the end of the local electrogram in sinus rhythm. The authors suggested that this might be due to a muscular connection between the pulmonary artery site and the RVOT. Subsequently, several investigators described the presence of the same discrete potentials in patients with PVCs from the outflow tracts in which the site of origin was above the semi-lunar valves. Tada et al [10] found sharp, local potentials, in 12 patients with RVOT ectopy originating above the pulmonary valve and Srivathsan et al [11] described discrete arterial potentials in all 12 patients with outflow tract ventricular arrhythmias, also originating above the semi-lunar valves.

However, the pre-systolic potentials described by those authors were only present when the origin of the PVCs was above the pulmonary valve. Thomsen et al [12] described, for the first time, the presence of similar discrete pre-systolic potentials in 24 patients with RVOT arrhythmias originating below the pulmonary valve. These pre-systolic potentials became late potentials during sinus rhythm, occurring at the end of the ventricular electrogram. According to Thomsen et al, they represent an area of conduction impairment, protecting an ectopic pacemaker by intermittent entrance block. We cannot rule out this mechanism in our patients, in fact, our diastolic potential may represent an ectopic focus protected by entrance block and intermittently capable of conducting to the adjacent myocardium. Supporting this hypothesis is the non-disappearance of the diastolic potentials after successful ablation in the majority of cases. This may suggest that we did not completely eliminate the focus, but instead may have created an exit block that rendered it incapable of propagating to a sufficient number of myocytes, in order to elicit a PVCs.

On the other hand, unlike the potentials described by Thomsen et al [12] our potentials occur late in diastole, after the end of the T wave, which corresponds to the phase 4 of the cardiac action potential [13].

Their occurrence during the phase 4 of the cardiac action potential suggests that they may result from delayed afterdepolarizations (DADs). This finding is in accordance with the generally accepted theory that the outflow tract ventricular tachycardia is caused by cAMP-mediated DADs and triggered activity [14]. The termination of the RVOT tachycardia in response to adenosine and to non-dihydropyridine calcium-channel blockers [15], along with its inducibility by rapid atrial/ventricular pacing or isoprenaline infusion, have been the clinical milestones for this theory. Other ventricular tachycardias that are thought to be due to triggered activity are the catecholaminergic polymorphic ventricular tachycardia and the ventricular tachycardia due to digitalis toxicity. The former is due to a mutation in the calcium ryanodine receptor gene (RyR2) or in the cardiac calsequestrin isoform 2 encoding gene (CASQ2) that leads to a cytosolic Ca^2+^ overload and DADs [16]. An inhibition of the Na^+^/K^+^-ATPase mediates the triggered activity due to digitalis toxicity [17].

Unlike these last two entities, in which the mechanism of the DADs is well known, in the RVOT tachycardia the precise mechanism for the occurrence of the DADs is not completely understood [18]. The demonstration of the presence of DADs in vivo has never been done, and the assumption that RVOT PVCs share the same mechanism of the RVOT tachycardia has not been proven. Kim et al [19] hypothesized that outflow tract arrhythmias may represent a continuum with increasing severity and a common mechanism. However, in their paper they did not prove such statement.

We speculate that our diastolic potentials may represent a form of triggered activity that results in a potential with a very low amplitude, only recorded when the catheter is in close proximity to their origin. If we consider that they are the source of the PVCs, it would be expected that their location would be at the site of successful RF application. When this potential is able to propagate to a critical number of adjacent myocytes it elicits the occurrence of the PVCs. That may depend on the intensity of the DADs or on the degree of exit block. A detailed mapping of the area is fundamental in order to find the earliest diastolic potential in relation to the beginning of the surface QRS. That may explain why in some RF applications the presence of diastolic potentials at the ablation site was not enough to ensure success.

The hypothesis that these potentials may represent an area of very slow conduction similar the ones implicated in the reentry circuit of scar related ventricular tachycardia [20], is unlikely. Firstly, because although we recorded the diastolic potentials in areas of low voltage in some patients, in others the area was completely normal. A second reason is that reentry is unlikely to be the mechanism of RVOT PVCs.

We did not find, in the literature, any other reports of diastolic potentials identical to ours, except for a clinical case published by Saha et al [21], in 2016. It describes a patient with Brugada syndrome and arrhythmic storm that underwent catheter ablation. The authors present an image with diastolic potentials very similar to ours but do not address it in the text.

Regardless of the mechanism for the occurrence of diastolic potentials, we strongly believe that they are the source of the PVCs. Their timing in relation to the ventricular electrogram inverts during the PVCs from being very late in sinus rhythm to very early. This fact, along with the finding that variations in the inter diastolic potential intervals lead to variations in the intervals between consecutive PVCs, suggests that they are related to the PVCs instead of being a bystander.

The second important finding of our study was the observation that the diastolic potentials were recorded mostly on areas of low voltage and fragmented electrograms, suggesting that the DADs occur in diseased areas.

It is usually accepted that in the absence of structural heart disease the intracardiac electrograms display normal duration and normal voltage [22]. However, we observed areas of low voltage in the majority of our patients even though the echocardiogram and cardiac magnetic resonance imaging did not demonstrate any form of structural disease.

The presence of these low voltage areas may be due to the thinner myocardial wall of the RVOT. The local electrograms in the septum area of the right ventricle have the highest voltage, as opposed to the ones from the RVOT, which display the lowest values [23]. Still, the normal accepted value for the bipolar electrogram amplitude in the RVOT area is normally above 1.5 mV. The presence of such low voltage electrograms in our patients supports the hypothesis that some forms of apparently idiopathic outflow tract PVCs/VTs may be substrate-related arrhythmias, as previously described [24,25].

Liu et al [24] have recently described the presence of low voltage electrograms at the successful ablation sites, suggesting that there may be a substrate-based mechanism for the RVOT arrhythmias. These authors also report the occurrence of discrete late potentials only present at the low voltage areas. The potentials described by Liu et al are similar to the ones described above the pulmonary valves, occurring within or shortly after the local ventricular electrogram in sinus rhythm. In a previous study [25] we also identified areas of low voltage in the electroanatomical mapping in some of the patients with apparently idiopathic RVOT premature ventricular contractions.

The definition of an idiopathic situation results from the absence of abnormalities in the diagnostic tests performed and from the lack of knowledge of the cause, but it does not imply absence of pathological abnormalities. This is the case with the Brugada Syndrome, that was assumed to be an electric disease without anatomical substrate, and yet, recently some authors demonstrated the presence of delayed fragmented potentials in the epicardium of the RVOT that were successfully ablated [26]. We describe, in our paper, the presence of low voltage areas and fragmented electrograms, however, the fragmented electrograms we describe in our patients are not like the ones present in Brugada Syndrome, which are characterized by being very late. In fact, our fragmented electrograms terminate before the end of the QRS and the long duration of the local electrograms are mostly due to the fusion between the low voltage diastolic potentials and the fragmented electrograms.

In our group of patients, low voltage areas and diastolic potentials were not always present. This may imply failure to identify the area of interest, but it can also mean that RVOT PVCs may have different mechanisms or substrates.

The presence of low voltage areas and diastolic potentials may be considered a new target for ablation. Detailed mapping of these areas is needed in order to find the earliest diastolic potential in relation to the surface QRS of the PVCs.

Substrate mapping to identify areas of low voltage and searching for diastolic potentials may be a possible ablation strategy for ablation of RVOT PVCs in patients in whom the clinical arrhythmia cannot be elicited.

## 5. Limitations

This study was retrospective, and the total number of patients included was small. One possible important information might have been obtained by pacing the area where diastolic potentials were recorded and assessing the response to pacing.

A prospective study including more patients and a control group to validate the role of diastolic potentials in guiding outflow tract PVCs ablation is needed. The primary ablation target would be the earliest diastolic potential.

## 6. Conclusions

Diastolic potentials were frequently recorded in idiopathic outflow tract PVCs. They were present mostly at low voltage areas, suggesting that outflow tract PVCs may have an anatomic substrate. Catheter ablation at sites with diastolic potentials is associated with an increased success rate. Substrate mapping to identify areas of low voltage and searching for diastolic potentials may be a possible ablation strategy for ablation of RVOT PVCs.

## Supporting information

S1 File De-identified patient database. (SAV)

S2 File De-identified radiofrequency applications database. (SAV)

## Abbreviations

PVCs: Premature ventricular contractions
RVOT: Right ventricular outflow tract
RF: Radiofrequency
ECG: Electrocardiogram
OR: Odds ratio
CI: Confidence interval
DADs: Delayed afterdepolarizations
